# Supplementary material for: Fostering empowerment: Transition from self-help groups to cooperatives in leprosy-affected communities in Nepal
Source: PLoS Negl Trop Dis. 2025 Dec 5;19(12):e0013799. doi: 10.1371/journal.pntd.0013799 (PMC12680263; doi:10.1371/journal.pntd.0013799)
Supplement: S1 File — Includes ethical approval letter from Nepal Health Research Council and ethics clearance details for the study (NHRC 444–2020 P). (PDF) [file pntd.0013799.s001.pdf]

Government of Nepal
Nepal Health Research Council (NHRC)
Estd. 1991

Ref. No.: 160.

26 July 2020

Dr. Indra Bahadur Napit

Principal Investigator

The Leprosy Mission Nepal, Anandaban Hospital, Lalitpur

Ref: Approval of research proposal

Dear Dr. Napit,

This is to certify that the following protocol and related documents have been reviewed and granted expedited from review by the Expedited Review Sub-Committee for implementation.

<table>
<tr><td>ERB Protocol Registration No.</td><td colspan="2">444/2020 P</td><td>Sponsor Protocol No</td><td>NA</td></tr>
<tr><td>Principal Investigator/s</td><td colspan="2">Dr. Indra Bahadur Napit</td><td>Sponsor Institution</td><td>University of Birmingham</td></tr>
<tr><td>Title</td><td colspan="4">Self Help Evaluation for lepRosy and other conditions in NePAl (SHERPA)</td></tr>
<tr><td>Protocol Version No</td><td colspan="2">Version 5.0</td><td>Version Date</td><td>18 July 2020</td></tr>
<tr><td>Other Documents</td><td colspan="2">1. Data collection tools
2. Donor agreement letter</td><td>Risk Category</td><td>Minimal risk</td></tr>
<tr><td rowspan="4">Expedited Review</td><td>Proposal</td><td>√</td><td rowspan="4">Duration of Approval
26 July 2020 to 26 July 2021</td><td rowspan="4">Frequency of continuing review
At least once in a year</td></tr>
<tr><td>Amendment</td><td></td></tr>
<tr><td>Re-submitted</td><td></td></tr>
<tr><td colspan="2">Meeting Date: 20 July 2020</td></tr>
<tr><td>Total budget of research</td><td colspan="4">NRs 2,73,26,074.80</td></tr>
<tr><td>Ethical review processing fee</td><td colspan="4">NRs 8,19,782.244</td></tr>
<tr><td colspan="5">Investigator Responsibilities
• Any amendments shall be approved from the ERB before implementing them
• Submit progress report every 3 months
• Submit final report after completion of protocol procedures at the study site
• Report protocol deviation / violation within 7 days</td></tr>
</table>

Tel: +977 1 4254220, Fax: +977 1 4262469, Ramshah Path, PO Box: 7626, Kathmandu, Nepal
Website: http://www.nhrc.gov.np, E-mail: nhrc@nhrc.gov.np

Government of Nepal
Nepal Health Research Council (NHRC)
Estd. 1991

Ref. No.: 160.

- Comply with all relevant international and NHRC guidelines
- Abide by the principles of Good Clinical Practice and ethical conduct of the research

If you have any questions, please contact the Ethical Review M & E Section at NHRC.

Thanking you,

Dr. Pradip Gyanwali
Executive Chief
(Member-Secretary)
