# Supplementary material for: Fostering empowerment: Transition from self-help groups to cooperatives in leprosy-affected communities in Nepal
Source: PLoS Negl Trop Dis. 2025 Dec 5;19(12):e0013799. doi: 10.1371/journal.pntd.0013799 (PMC12680263; doi:10.1371/journal.pntd.0013799)
Supplement: Attachment — Submitted filename: Response to reviewers.docx [file pntd.0013799.s005.docx]

Date: 4^th^ September 2025

Dear editor and reviewer,

Thank you very much for your detailed and constructive evaluation, and opportunity to revise our work. We have followed each of your suggestions to improve the manuscript. Please see below our responses to your specific suggestions with corresponding changes in the manuscript.

We look forward to your kind consideration.

Sincerely,

Dilip Shrestha

1. While the study provides rich descriptive material, the methodology is weakened by the absence of a clearly articulated research question or a set of specific, pre-defined objectives guiding data collection and analysis. The work unfolded over several years as part of an ongoing programme, but the design lacks a formal temporal framework or a structured plan for how observations and interviews would be sequenced, compared, or integrated over time. This long-term, open-ended engagement risks blurring the line between programme implementation and research, making it difficult to assess the consistency of data collection or the influence of evolving programme priorities on the findings. Without a clearly stated guiding question and analytical boundaries, the study functions more as an extended narrative account of programme activities and perceived impacts than as a systematically designed research investigation.
That being said, it could easily be improved by providing more transparency on the specific methodological design that was intended before the start of the study or justification for a narrative design, but with the addition of more information on when and over how much time the IDI and FGD took place.

The manuscript claims a “mixed-methods” approach but lacks meaningful integration of the quantitative aspect. The analysis of the interview transcript through content analysis does not make it a quantitative approach. Therefore, it reads like a qualitative methodology, and the mention of “mixed methods” should be removed.

AUTHORS: Thank you for your question. We have elaborated study design, and it read as,

This study employed a mixed method design with elements of quantitative and qualitative research. First, a cluster-based, non-randomised controlled study evaluated the effectiveness of self-help groups on ulcer metrics (people affected by leprosy only) and on four generic outcome measures (all participants) - generic health status, wellbeing, social integration and household economic performance. Second, a qualitative study aimed to examine the implementation and sustainability of the intervention. This article involves a deeper exploration of findings from qualitative data, incorporating those from focus group discussions, in-depth interviews, and key informant interviews from participants such as people affected by leprosy, single women, community stakeholders and facilitators.)

2. Explain where and for how long you will store the collected data. For your information: The PLOS Data policy requires authors to make all data underlying the findings described in their manuscript fully available without restriction, except in cases where the data are legally or ethically restricted (for example, participant privacy is an appropriate restriction).

Limitations could be moved to the discussion section and explained in text form instead of bullet points.

AUTHORS: Thank you for your suggestions. We have moved the limitations to the discussion section and read as,

A limitation of our study is that the in-depth interviews participants were selected using a random sampling technique, which may not have adequately captured the lived experiences. Additionally, since the interviews were not conducted in the local language, there is possibility of misinterpretation or limited depth of responses.

All data collected for this study, including transcripts and field notes, are securely stored in password-protected files on institutional servers at The Leprosy Mission Nepal. In line with Nepal Health Research Council (NHRC) ethical requirements, the data will be preserved for a minimum of five years after publication. Due to the sensitive nature of the information and the risk of participant identification, the full dataset cannot be made publicly available.

Data Saturation needs elaboration. The authors briefly mention achieving saturation but provide limited detail. Explain how saturation was assessed across different participant types (e.g., SHG members vs facilitators vs stakeholders). However, the concept of data saturation in qualitative research is being debated. If the authors do not have a convincing argument for the achievement of data saturation, it can also be left out to not provide room for uncertainty.

AUTHORS: Thank you for your suggestion. We have elaborated this part and read as follow

Data saturation was achieved through the triangulation of multiple data sources, including interviews with diverse participants. The interview contents were reviewed and categorised across project beneficiaries, community-based stakeholders and project facilitators on ‘Self-care’, ‘SHG’, ‘Cooperatives’, and ‘Sustainability’. By incorporating the perspectives of project beneficiaries, community-based stakeholders and project facilitators, we ensured saturation when that no new themes or insights emerged during analysis.

Ethical Considerations need strengthening. While ethics approval is mentioned, there is insufficient detail on how confidentiality, translation, and interviewer–interviewee dynamics were handled in sensitive contexts. The authors could provide information if written consent was given by the interviewees after the explanations, and if not, provide a rationale. Moreover, they could expand on how interviews were conducted ethically, particularly regarding social stigma and power imbalances.

AUTHORS: Thank you for your suggestion. We have added the context regarding written context and translation and read as follow,

A patient information leaflet and consent form translated in local language and back translated according to the WHO methodology was given to the participants. A written consent was obtained before the interview. Measures were taken to ensure that individuals felt comfortable and did not experience feelings of inferiority. Questions were posed thoughtfully, using appropriate terminology to respect the experiences of participants affected by leprosy, living with disability and those marginalized.

3. The methods used in this study, FDG, IDI and KII are appropriate to generate qualitative information to attain to the objectives of this research. The objectives stated in the introduction seem to address the whole mixed-methods study that was conducted. Since this paper solemnly covers the qualitative part, the objectives would benefit from a more precise formulation of the research question that were relevant for the qualitative part. Formulating the questions more precise would greatly benefit the reader in understanding the relevance of the rich results and discussion this study produced. The research population and sampling strategies are described in detail, in both the “study setting” and the “sampling and participants” paragraphs. For more clarity, these two could be re-designed since information on the participants is also in the “study setting”. Data collection, saturation (incl. the sample size n=118) and analysis are clearly written and reasoned, giving a nice overview of the workflow that has been conducted. The ethical clearance was adequately obtained, and measures to ensure confidentiality and eye-level interviews are succinctly described.

The only thing I am wondering is if written consent was obtained by all the participants of this study? If so, it would be good to state that explicitly in the “Ethics”. The limitations mentioned in the methods section could be placed in the “Discussion” to be discussed for the impacts on the results and further studies.

AUTHORS: Thank you for your remark. Yes, this study is solely a phenomenological observational approach, and the analysis is not purposefully based on previous theoretical works.

4. Reviewer #3: The results describe the successes/benefits and challenges of the measures implemented through the IMPACT project. The four key themes, self-care, SHG, cooperatives and sustainability arose from interview analysis. The results are very much presented in detail with additional context and are very well and understandably written. Under the theme of “Sustainability” the authors describe the role of family and community involvement in leprosy, and additionally the role of government actions in leprosy awareness and difficulties with leprosy awareness campaigns. This should be ideally reflected in a separate title – as it rises important new points separate from family and community involvement.

AUTHORS: Thank you for your insight. However, we believe that incorporating the content as proposed would risk omitting a significant portion of the ‘sustainability’ theme. We feel it is most appropriate to present them under same theme.

Generally, some of the interview quotes feel lengthy, and could benefit from a little shortening to emphasize and support the results better.

AUTHORS: Thank you for your suggestion. We agree with your point about lengthy interview quotes, and we have shortened the quotes.

Conclusions

5. Reviewer #1: The conclusions are well-supported by the data, and the public health relevance of the research is strong and can provide a great model for mixed self-help groups in the future. I had one suggestion for the authors to include additional information about the limitations associated with languages spoken by participants and researchers.

In the limitations section, the authors write, “Interviews were not conducted in the local language.” Could you say a little more about this? Were they in Nepali, and if so, what is the level of fluency of people for whom that is a second language or the language of school vs. what is spoken at home? What was the local language? Do you feel like this was an issue more for people in self-help group participants than for facilitators or other community leaders?

AUTHOR: Thank you for your question. As the people from the region speaks local languages such as Bhojpuri, Awadhi, Tharu and Maithili to lesser extent but it was resolved by hiring local project officer.

6. Reviewer #2: Provide a clearer statement of how this study addresses the public health relevance and a recommendation for future research.

AUTHOR: Thank you for your question. It has been addressed.

7. Reviewer #3: The discussion very well summarizes the results and their significance and is very well composed. The following paragraphs discuss the implications that self-help groups have on the management of the different impacts of leprosy. It very well contextualizes the findings from the study with results from similar studies from other countries and contexts. For a more precise understanding maybe it would be beneficial to shortly outline the contexts of the studies that are mentioned, e.g. were these also studies on SHG? Were these conducted with people affected by leprosy, or other marginalized groups as well? Did you have findings that other studies did not have? Or any contradictory points? This could be worth discussing as well if there were some.
Limitations are mentioned in the methods of this manuscript. Personally, I’d suggest moving and discussing them here, as it allows to discuss the implications of the limitations for this and further research on this topic.
Overall, the discussion does very well embed the findings from this study into the broader context, emphasizing its significance and the long-term effects. The discussion part on sustainability and the need for community-led interventions demonstrates the transferability of the findings into possible other contexts as well – highlighting its relevance for public health and public health research.

AUTHOR: Thank you for your question. We have revised the discussion section and incorporated the context as suggested.

Use this section for editorial suggestions as well as relatively minor modifications of existing data that would enhance clarity. If the only modifications needed are minor and/or editorial, you may wish to recommend “Minor Revision” or “Accept”.

Reviewer #1: Here are a few minor editorial suggestions:

p. 4, lines 8-9: “Leprosy is closely linked to poverty and economic hardships because individuals with the disease often experience stigma and discrimination, beyond their physical deformities.” I would restate this to say that something about the complex relationship between leprosy and poverty, with some of the conditions of extreme poverty contributing to spread of the disease and the disease itself resulting in stigma and discrimination, which can result in or further exacerbate economic marginalization.

p. 4, lines 14-15: Explain the criteria for “elimination” as used by WHO, since it is different from the dictionary definition of the word. It might be helpful to include the latest numbers of incidence and prevalence in Nepal here too.

p. 4, line 23: Can you include a little more information about IMPACT and how it is funded? Is it an NGO?

p. 7, line 21: “facilitators”—maybe say “facilitators of the self-help groups” (or does this mean overall project facilitators)?

p. 11, line 9: “member’s” should be “members’”

p. 22: In the quote about planting sunflowers (DI, P04, Male, PLWD), is he saying the loan was not enough because it didn’t cover the cost of pesticides and fertilizers? Maybe a sentence of explanation between quotations here could be useful.

Reviewer #2: Double-check the manuscript for language and grammar issues, consulting a native English speaker or a tool.
The abstract should include specific numbers or key statistics (e.g., number of participants, number of groups).
Double-check that acronyms are clarified on their first use (e.g., PLWD).

AUTHOR: Thank you so much for your observation, we have clarified the acronym and read as follow,

People living with disability (PWLD)

**Summary and General Comments**


Use this section to provide overall comments, discuss strengths/weaknesses of the study, novelty, significance, general execution and scholarship. You may also include additional comments for the author, including concerns about dual publication, research ethics, or publication ethics. If requesting major revision, please articulate the new experiments that are needed.

Reviewer #1: This is an excellent article on what seems to be a very promising model for how self-help groups can be successful and sustainable. You all do a great job demonstrating a self-help group with mixed participation from groups of different marginalized statuses and identities can help each other, and you provide a good discussion of some of the logistical challenges that were faced.

You note that a benefit of a mixed self-help group that includes people affected by leprosy is that it can help to destigmatize leprosy. I found this also in observing an existing discussion group that was held in a general dermatology treatment center in Brazil; there were people coming in for many other conditions, but a social worker would lead discussions about leprosy, and this led to people bringing up their misconceptions and learning new things. You mentioned that more affluent or higher caste people affected by leprosy may not want to attend a group that is leprosy-specific, but were they more likely to attend when the group was expanded to include other marginalized groups?

AUTHOR: Thank you for your insight. However, exploring whether the affluent or high-class people affected by leprosy would be willing to attend a leprosy specific group was beyond the scope of our study.

Reviewer #2: Dear authors of PNTD-D-25-00023,

Thank you for a highly relevant and original study on the transition from Self-Help Groups (SHGs) to cooperatives within the context of leprosy-affected and marginalised communities in Nepal under the IMPACT initiative. The manuscript addresses an important and under-researched issue in neglected tropical diseases (NTDs), particularly the intersection of social empowerment, stigma, and sustainability in leprosy-affected communities. The shift from SHGs to cooperatives is a novel angle, showing a long-term development trajectory beyond health.
The study design includes focus group discussions (FGDs), in-depth interviews (IDIs), key informant interviews (KIIs), and observation, providing data triangulation and depth. Moreover, the use of COREQ guidelines demonstrates rigour and transparency in qualitative research.
The narrative quotes provide powerful, real-life illustrations of transformation in the lives of marginalised individuals. The cultural and gender-sensitive findings are well-articulated.

Reviewer #3: Introduction: The introduction gives a clear outline on the topic of leprosy in Nepal, the sociocultural implications of leprosy and the role of self-help and -care. The topic of the paper is well introduced. Moreover, it introduces the reader to the IMPACT study. This part of the introduction could benefit from some examples on what the interventions within the IMPACT project look like to better contextualize the findings from the results. For additional context, it might be beneficial to embed the study in a summary of some pre-existing literature, that seem highly relevant or even linked to the study, such as Pryce et al. 2018 (Assessing the feasibility of integration of self-care for filarial lymphoedema into existing community leprosy self-help groups in Nepal) or Shresta et al. 2021 (Evaluation of a self-help intervention to promote the health and wellbeing of marginalized people including those living with leprosy in Nepal: a prospective, observational, cluster-based, cohort study with controls).

AUTHOR: Thank you very much for your suggestion.

General Comments: The findings of this study are very interesting, and seem to depict an innovative, sustainable and long-term approach to address marginalization and stigmatization of people affected by leprosy and other marginalized groups. Overall, the manuscript offers new insights relevant to researchers and policy makers not only in Nepal but also in other settings. The main points to address before publication revolve around the clearance of the objectives and research questions, to provide the reader with a clear outline of this research, and the contextualization of the research itself (in the introduction) and the contextualization of the results in the discussion part. Considering the results describe successes and challenges of the measures implemented in the IMPACT project – maybe this should reflect in the title (and the research question)?

AUTHOR: Thank you for your suggestion. We have referenced the study by Shrestha et.al. 2021.
