# Supplementary material for: Fostering empowerment: Transition from self-help groups to cooperatives in leprosy-affected communities in Nepal
Source: PLoS Negl Trop Dis. 2025 Dec 5;19(12):e0013799. doi: 10.1371/journal.pntd.0013799 (PMC12680263; doi:10.1371/journal.pntd.0013799)
Supplement: Attachment — Submitted filename: Response_to_Reviewers_auresp_2.docx [file pntd.0013799.s006.docx]

Date: 3^rd^ November 2025

Dear editor and reviewer,

Thank you very much for your detailed and constructive evaluation yet again, and opportunity to revise our work. We have followed each of your suggestions to improve the manuscript. Please see below our responses to your specific suggestions according to each reviewer. We look forward to your kind consideration.

Sincerely,

Dilip Shrestha

**Reviewer #1**

**Feedback:**

**Key Review Criteria Required for Acceptance?**
As you describe the new analyses required for acceptance, please consider the following:

**Methods**

-Are the objectives of the study clearly articulated with a clear testable hypothesis stated?

-Is the study design appropriate to address the stated objectives?

-Is the population clearly described and appropriate for the hypothesis being tested?

-Is the sample size sufficient to ensure adequate power to address the hypothesis being tested?

-Were correct statistical analysis used to support conclusions?

-Are there concerns about ethical or regulatory requirements being met?

AUTHORS: Thank you for your Queries. We have corrected in the method section and abstract as well.

While the broader study adopted a mixed-methods design integrating both quantitative and qualitative approaches, the present article is based exclusively on the qualitative data used for the analysis.

The details are in the ethical section.

We were thoughtful of the participants enrolled in our study. We followed following process for the patient privacy and confidentiality. A patient information leaflet and consent form translated in local language and back translated according to the WHO methodology was given to the participants. A written consent was obtained before the interview. Measures were taken to ensure that individuals felt comfortable and did not experience feelings of inferiority. Questions were posed thoughtfully, using appropriate terminology to respect the experiences of participants affected by leprosy, living with disability and those marginalized.

**Reviewer #2**

Clarity of Research Question:
The Introduction is much improved, but please include a clear, single-sentence research question or aim at the end of the section to sharpen the study’s focus.

To what extent do self-care practices, self-help groups, and cooperatives improve the long-term sustainability of community-based leprosy management programs?

Study Design Terminology:

The manuscript is primarily qualitative, though part of a larger mixed-methods study. Consider rephrasing to emphasize that this paper reports the qualitative component of that broader design.

AUTHORS: Thank you for the suggestion. We have changed it according to your suggestion.

Data Saturation Description:
Clarify how data saturation was determined (e.g., no new codes/themes emerged after a certain number of interviews or groups).

AUTHORS: Thank you for your suggestions. Here is our response.

Interviews were conducted until data saturation was attained, defined as the point at which additional data failed to generate new codes or add meaningful variation to the existing themes.

Conciseness of Results:
The Results section is rich in participant narratives but remains lengthy. Some repetitive quotations could be reduced without losing the depth of insight. Aim for clearer synthesis across the four major themes.

AUTHORS: Thank you for the feedback. We have tighten the result by reducing the redundant contents. It is available in the “Revised article with highlighted changes”.

**Reviewer #3**: To improve quality, I would suggest avoiding explaining the mixed-methods approach, since the presented research in this article is solemnly qualitative.

AUTHORS: Thank you for the suggestion. We have rephrased it.

**The WHO methodology used for the interviews needs referencing.**

AUTHORS: Thank you for it. We had referenced it.
World Health Organization. Process of translation and adaptation of instruments. Geneva: World Health Organization; 2016

**Reviewer #4**:

The Introduction and Methods part of the abstract do not clearly enough correspond to the qualitative study described in the article but rather refer to the broader IMPACT initiative. This could mislead the reader. Please revise the abstract to reflect better the qualitative component and clearly distinguish between the overall IMPACT programme and this specific study.

AUTHORS: Thank you, we have changed according to your suggestion.

For introduction:
Although leprosy is curable, many affected individuals continue to face disability, stigma, and poverty. In Nepal, sustaining progress after elimination remains challenging, making community-based approaches such as self-care and self-help groups crucial for long-term health and social inclusion. This study investigates how self-help groups and cooperatives contribute to sustaining community-driven leprosy management efforts.

Methods:

For this study only qualitative data are utilized which involves people affected by leprosy, single women, community stakeholders, and facilitators. Conventional content analysis was employed to examine the data, focusing on participants' experiences and perceptions.
Similarly, the objective appears to be linked to the full IMPACT study. Objectives referring to the qualitative study should be stated especially considering that a clear, guiding research question is missing.

AUTHORS: Thank you, we have changed according to your suggestion.

Objectives

The study aims to to explore how self-care practices and the self-help group transition to cooperatives in enhancing the sustainability of community-driven leprosy management programs.

Question:

This research tends to understand the extent do self-care practices, self-help groups, and cooperatives improve the long-term sustainability of community-based leprosy management programs.

The introduction could be strengthened by a more thorough discussion of the existing literature and an explicit emphasis on what is unknown and what gap you want to fill. What question are you answering? Reading the title, one expects something along the lines of ‘What are the experiences and perceptions of key stakeholders (people affected by leprosy, facilitators, community leaders...) regarding the IMPACT initiative to sustain community-based leprosy programmes. Later, it sounds more like: "What are the experiences and perceptions of key stakeholders in the IMPACT initiative (people affected by leprosy, facilitators, community leaders...) regarding the successes and challenges of the IMPACT programme (the role of self-care, participation in self-help groups) in promoting health equity, disease control and socio-economic empowerment, as well as cooperative transformation in sustaining community-based leprosy programmes?" The guiding research question should be clear and consistent from the title to the conclusion.

Methods
Although most of the important information is included, the Method section would benefit from some of the Information being reorganized and allocated more fittingly into the different subsections.

Study design - P 5: Consider starting the paragraph of “study design” with “The larger IMPACT study employed a mixed-method design…..” to make the delineation between full IMPACT and this qualitative study clearer.

AUTHORS: Thank you, we have changed according to your suggestion.

Study setting - Quite clear. The differentiation between the IMPACT programme implementation area and the sub-areas considered in this study could be a bit clearer. The Description of study population belongs in the participant section

AUTHORS: Thank you, we conducted in same area.

Participants and Sampling – What were the inclusion and exclusion criteria, the target group and the reasons for the sampling strategy. The rationale for using a random rather than a purposive sampling technique should be explained. The description of data collection methods (IDI, KII…) belongs to the data collection section. On page 6 you mention “a checklist based on the framework to record behaviour change in this section”– what do you mean? Is this a theoretical framework that guided methodology/analysis? If so this would make more sense in the introduction and/or study design.

AUTHORS: Thank you for your feedback. Here is our response. It is available in “Revised article with highlighted changes”.

A random selection of 6-10 group members were invited to participate in the discussion without the facilitator present. Random selection helped capture a broader range of experiences- including those who may be less confident, less visible, or less involved in group activities- thereby strengthening the validity and balance of data. The participants were included from the self-help group members and those who did not consent for the interview were not included in the study.

Data collection - Summarise how, when, and by whom the data was collected, including the instruments (interview guides), duration, and logistics of the sessions. Many parts currently mentioned in the participant section would be better placed here.

AUTHORS: Thank you for the suggestion. We have rephrased.

The first author (DS) and research officers (SA, SS, AS, SP^1^, DT, SP^2^, PA) conducted in-person interviews. The FGD, key informant interview (KII) was conducted by the first author. DS is a project Hub Manager and currently a DrPH Scholar at Mahidol University, previously working on the project [9], which was led by co-author Indra Bahadur Napit (IBN), who has extensive experience in the field of leprosy and is currently working at The Leprosy Mission Nepal (TLMN). Fieldwork began in July 2021, and data analysis was completed by May 2024. The piloting of the topic guide was conducted in a similar community and group of up to 10 individuals to ensure consistency across sessions. Formal interviews and group discussions were conducted only once as per the ethics using a topic guide, with each session lasting between 30 to 90 minutes. Audio recorders were used to record the interviews and discussions with prior consent. Since this research was part of an evaluation, the program team was stationed at the field site, helping to build relationships and rapport with the participants.

Data analysis - This section is clear.

Ethical considerations - This section needs to be strengthened.
1. You mention that measures were taken to ensure that people felt comfortable (p 8) – what were these measures? As some of the participants belong to vulnerable groups please include that measures to minimise harm and ensure psychological/emotional safety were taken, for example, accessible venues for FGDs and interviews, as some participants had disabilities.

2. Voluntary participation and voluntary withdrawal should be explicitly mentioned, as should measures to avoid coercion – especially as this statement “They described their efforts to convince people to join these groups despite facing significant resistance, emphasizing a personal commitment to social change” (p 13) raises questions about social coercion. What were the efforts to persuade people to participate, and how did you avoid coercion?

AUTHORS: Thank you for your feedback and we have edited according your suggestion.
We have answered it
The purpose of the study was explained to potential participants either during a group orientation session or individually. This study received ethical approval from the Nepal Health Research Council on NHRC 444-2020 P. The ethical clearance document is attached with (Supplementary 1). To ensure participants felt comfortable and safe, interviews and FGDs were held in physically accessible, familiar community settings and scheduled at times chosen by particiapnts. Participation was entirely voluntary, and individuals were informed that they could decline to answer any question, take breaks or withdraw from the study at any stage without any consequnce. Privacy was maintained throughout. A patient information leaflet and consent form translated in local language and back translated according to the WHO methodology was given to the participants. A written consent was obtained before the interview. The research team was trained in disability-inclusive and trauma-sensitive interviewing, enabling them to respond appropriately to emotional discomfort and provide referral information when needed. Participants were informed that they could skip any question or withdraw at any point without consequence. A supportive environment was maintained by ensuring privacy, using local language, adopting non-judgmental communication, and having same-gender interviewers where preferred. Questions were posed thoughtfully, using appropriate terminology to respect the experiences of participants affected by leprosy, living with disability and those marginalized.

All data collected for this study, including transcripts and field notes, are securely stored in password-protected files on institutional servers at The Leprosy Mission Nepal. In line with Nepal Health Research Council (NHRC) ethical requirements, the data will be preserved for a minimum of five years after publication. Due to the sensitive nature of the information and the risk of participant identification, the full dataset cannot be made publicly available.

The results are comprehensive, rich in qualitative detail and underpinned by well-chosen quotes from participants. However, the absence of a clearly formulated research question or guiding framework sometimes makes it difficult for the reader to follow the thread.

AUTHORS: Thank you for your suggestion and we have addressed.

Regarding figures:
- Page 7: you refer to Supplementary table 1) for information on the 80 participants participating in the FGDs. Consider also adding the characteristics of the FDG participants (gender, age, condition etc.) to Table 1 for clarification and completeness.

AUTHORS: Thank you for your suggestion and details are in the other tables.

- The map is currently not sufficient in quality. The font is too small to be legible and cannot really be assessed.

AUTHORS: Thank you and we have re-shaped it.

- Supplementary Table 1 lists a 106-year-old participant - please verify that this information is correct.

AUTHORS: Thank you for queries. It is confirmed.

Consider including as a limitation the exclusion of non-participants, as those who declined or dropped out may have offered important contrasting insights

AUTHORS: Thank you for your suggestion.
